# Post-Mortem Effects as a Variation Factor in *Lachesis muta* Venom

**DOI:** 10.3390/toxins18070288

**Published:** 2026-06-30

**Authors:** Mariana Silva Tavares, Melissa Inácio Moitas, Caroline Serino-Silva, Savio Stefanini Sant’Anna, Kathleen Fernandes Grego, Anita Mitico Tanaka-Azevedo

**Affiliations:** 1 Laboratory of Herpetology, Butantan Institute, São Paulo 05503-900, Brazil; mariana.tavares@usp.br (M.S.T.); melissamoitas@usp.br (M.I.M.); caroline.silva@fundacaobutantan.org.br (C.S.-S.); savio.santanna@butantan.gov.br (S.S.S.); kathleen.grego@butantan.gov.br (K.F.G.); 2 Interunit Postgraduate Program in Biotechnology, University of São Paulo, São Paulo 05508-000, Brazil

**Keywords:** *Lachesis muta*, post-mortem analyses, snake venom

## Abstract

Snakebite incidents could occur even with deceased animals due to improper animal manipulation, since the venom may retain biochemical activity. However, it is unclear what alterations death may cause in snake venom, because even in well-preserved genera, such as Lachesis, venom is susceptible to variation due to several factors. Therefore, the purpose of this research is to compare *Lachesis muta* venom characteristics before and after its death. The investigation encompasses protein profile analyses, immune recognition by antivenom, and enzymatic and biological activities. Venom samples were collected from the same individual at three distinct time points: two samples while the specimen was alive and a sample post-mortem. In RP-HPLC, a shift in peak intensity was observed in the peptide and protease elution regions. The post-mortem sample presented more PLA_2_ and LAAO enzymatic activities and a decrease in the coagulation capacity. All samples were well immuno-recognized by anti-*Bothrops*/*Lachesis* serum. Data suggest that *L. muta* venom maintains its functional and immunological integrity throughout senescence and following the animal’s death. The preservation of its biochemical properties supports the clinical relevance of accidents involving dead snakes and justifies the inclusion of such venoms in antivenom manufacturing protocols, providing a strategic alternative during supply shortages.

## 1. Introduction

The genus *Lachesis* belongs to the Viperidae family and is represented in Brazil by two species: *Lachesis muta*, found in the Amazon region, and *Lachesis rhombeata*, endemic to the Atlantic Forest [1,2]. Known as surucucu or surucucu-pico-de-jaca [3,4], *Lachesis* is one of the most medically important genera in the North and Northeast of Brazil. In 2024, according to SINAN, 24,962 cases of snakebites involving venomous snakes were reported in Brazil. The genus *Lachesis* was accountable for 392 of these incidents, representing 1.57% of the total in the country [5]. The clinical symptoms of *Lachesis* accident include pain, edema, blisters, necrosis, coagulation disorders, and cardiovascular disorders. The patient can also experience vagal syndrome, consisting of abdominal cramps, vomiting, diarrhea, dizziness, blurred vision, loss of consciousness, and sweating [3,6,7,8]. These symptoms are caused by the proteins that compose the snake’s venom, which constitutes 90% of their dry weight [9]. In the surucucu venom, the main protein families found are metalloproteinases (SVMPS), serine proteases (SVSP), phospholipases A_2_ (PLA_2_), L-amino acid oxidase (LAAO), among other enzymatic and non-enzymatic proteins. As well as a significant portion of peptides, such as C-type natriuretic peptide (CNP) and bradykinin-potentiating peptide (BPP) [10,11,12,13,14].

Surucucu is a nocturnal snake that usually hides in forests, avoiding contact with humans [3,8]. The maintenance and breeding of this species in captivity is challenging, which leads to difficulties in studying its venom [3,10]. In this context, the Butantan Institute has, throughout the years, improved the husbandry of venomous animals ex situ, updating and improving protocols that ensure a better quality of life for these animals and increasing their life expectancy [15]. At the Herpetology Laboratory, it is possible to acquire unusual samples, including the venom of recently deceased animals, such as the case with the specimen of *Lachesis muta*, which died from natural causes (heart attack) and had its venom collected two days after its death.

The protein content of venoms from the *Lachesis* genus is considered to be well preserved between species [11]. However, there are factors that can influence the variability in the protein content of snake venoms, such as sex, geographic distribution, seasonality, diet, and age [16,17,18,19,20,21,22,23,24]. For the genus *Bothrops*, there are several studies on venom variation throughout the snake’s development, focusing on the ontogeny of the animal [18,25,26,27], as well as studies on the impact of age in the *Lachesis* genus [1,11,28]. Similarly, research studies focusing on the venom of aging animals are beginning to gain attention, shedding light on the significant influence of age on the composition and potency of snake venom [29,30,31]. Nevertheless, the potential impact of post-mortem physiological changes on the biochemical composition and functional properties of snake venom remains an underexplored area of research. Considering this research gap, the present study aimed to analyze whether death influences the variation in the venom of a *Lachesis muta* specimen by comparing three samples from the same animal. Two samples were milked while the snake was still alive, in 2015 and 2023, and the last one after its death, in 2024, to analyze enzymatic and biological activities, protein profile, and immunorecognition by antivenom serum.

## 2. Detailed Case Description

### 2.1. Venoms and Antivenom Serum

The venom evaluated is from a female *Lachesis muta* from nature—Rio Branco (AC—Brazil). This animal was incorporated into the Herpetology Laboratory at the Butantan Institute (HL-BI) in 2015, measuring 176 cm in total length and weighing 1.885 kg, equivalent to a surucucu approximately 4 years old. The snake lived for 9 years in captivity, reaching an estimated total lifespan of roughly 13 years. Three samples from the same animal were analyzed: Lm_15, corresponding to the venom extracted when the serpent arrived at the serpentarium of HL-BI in 2015; Lm_23, from the last extraction of the live animal destined to produce antivenom serum in 2023; Lm_24, venom extracted two days after the animal’s death in 2024. Manual extraction of the samples was performed according to the standardization established by the HL-BI [15]. The extracted venoms were centrifuged at 1700× *g* for 15 min, lyophilized, and stored at −20 °C until analysis.

The commercial anti-*Bothrops*/*Lachesis* serum (ABLS, batch: 210185) was supplied by the Butantan Institute. This antivenom is produced using purified antibodies from the plasma of horses hyperimmunized with a sublethal dose of snake venom. As a mixed serum, ABLS is the result of combining antibodies produced by inoculation with a botropic pool (50%), composed of venoms from *Bothrops jararaca* (50%), *Bothrops jararacussu* (12.5%), *Bothrops alternatus* (12.5%), *Bothrops moojeni* (12.5%), and the *Bothrops neuwiedi* complex (12.5%), and antibodies produced from the inoculation of *Lachesis muta* and *Lachesis rhombeata* venom (50%) [32].

### 2.2. Protein Profile

Protein electrophoresis was performed according to Laemmli [33]. Venom samples were subjected to gradient polyacrylamide gel (10 to 20%) under reduced (with β-mercaptoethanol) and non-reduced conditions. Twenty micrograms of protein were added to each lane. The Dual Color Precision Plus protein standard was used as a molecular weight parameter, and the gel was stained with Coomassie Blue G250.

Reverse-phase high-performance liquid chromatography (RP-HPLC) followed the protocol described by Gay [34]. One milligram of lyophilized venom was resuspended in 1 mL of solution A (0.1% trifluoroacetic acid) and centrifuged at 13,000 rpm for 10 min. The supernatant (500 µL) was applied to a C-18 25 × 0.4 mm reverse-phase column (Teknokroma Europa 300, Sant Cugat del Vallés, Spain) previously equilibrated with 5% of solution B (95% acetonitrile and 0.1% trifluoroacetic acid) and 95% of solution A using the ÄKTA Purifier UPC10 chromatograph (GE Healthcare, Chicago, IL, USA). The samples were eluted according to the gradient of solutions A and B: isocratic (5% B) for 5 min, followed by 5 to 25% B for 10 min, 25 to 45% B for 60 min, and 45 to 70% B for 10 min. The flow rate was 1 mL/min, and absorbance was monitored at a wavelength of A215 nm.

### 2.3. Immunoassay

Western blotting was performed based on an electrophoresis gel and followed the protocol of Harlow [35]. Following SDS-PAGE using a 10–20% gradient polyacrylamide gel, proteins were transferred onto a PVDF (polyvinylidene difluoride) membrane, which had been previously equilibrated in transfer buffer (25 mM Tris, 192 mM glycine, and 20% ethanol). The membrane was then placed in a blocking solution (5% milk powder and 0.01% Tween 20) overnight at 16 °C with agitation. After blocking, the membrane was washed three times with washing buffer (10 mM Tris buffer, pH 7.5, with 150 mM NaCl and 0.01% Tween 20) and then placed in incubation buffer (5% powdered milk and 0.01% Tween 20) with primary antibody at a ratio of 1:1000 (ABLS; batch: 210185) for 2 h with agitation at room temperature. After three more washes, incubation with secondary antibody at a ratio of 1:10,000 with anti-horse antibody (batch: #058M4783V) was performed for 2 h with agitation at room temperature. The membrane was washed three times before adding the developing solution containing DAB (diaminobenzidine), H_2_O_2_, 0.1 M imidazole buffer (pH 7.0), and CoCl_2_.

The indirect ELISA test evaluated the ability of *L. muta* venom to be recognized by ABLS and was based on Harlow [35]. The 96-well plate was coated with 100 µL of a 10 μg/mL antigen solution (L. *muta* venom) in carbonate buffer (Na_2_CO_3_ 34 mM, NaHCO_3_ 15 mM, pH 9.6) overnight at 4 °C. After antigen fixation, the blocking solution (3% non-fat dry milk) was added for 2 h at 37 °C. After the second incubation, a serial dilution of 1:40,000 of ABLS (batch: 210185) in incubation buffer (1% non-fat dry milk, 0.05% Tween 20) was applied, and the plate was incubated again for 1 h at 37 °C. The immunoenzymatic conjugate (anti-horse IgG conjugated with peroxidase; batch: #058M4783V) was applied diluted in incubation buffer at a ratio of 1:10,000 and incubated for another 1 h at 37 °C. The washing process was performed between each step with a 0.77 M NaCl buffer with 1.25% Tween 20. Next, the substrate (OPD (o-phenylenediamine) 1 mg/mL, 1% H_2_O_2_) was diluted in citrate buffer (pH 5.0), applied, and incubated at room temperature for 5 min. After this time, the reaction was stopped with a 30% sulfuric acid solution. The plate was read on a SpectraMax i3 plate reader (Molecular Devices, San Jose, CA, USA) at 492 nm.

### 2.4. Enzymatic Activities

The enzymatic activity of L-amino acid oxidase (LAAO) followed the methodology of Kishimoto and Takahashi [36]. In a 96-well plate, 10 μL of venom (1 mg/mL) diluted in 0.85% saline was incubated with 90 μL of reactive solution containing 2 mM OPD (o-phenylenediamine), 250 mM L-methionine, 0.8 U/mg horseradish peroxidase, and 50 mM Tris-HCl buffer, pH 8.0. Saline was used as a negative control, and a standard hydrogen peroxide curve was applied as a parameter to determine the LAAO enzyme activity of the samples. The plate was incubated for 60 min at 37 °C, the reaction was stopped by adding 50 μL of 2 M H_2_SO_4_, and the reading was performed in an Epoch plate reader at 492 nm. The activity of LAAO was expressed as mM/min/mg and is based on the release of H_2_O_2_ in mM per incubation time per mg of protein compared to the hydrogen peroxide standard curve.

Hyaluronidase (HYA) activity was measured as described in Pukrittayakamee [37] with modifications. Ten microliters of venom (1 mg/mL) diluted in 0.85% saline, 20 μL of hyaluronic acid (0.5 mg/mL) diluted in sodium acetate-acetic acid buffer, and 70 μL of sodium acetate-acetic acid buffer (0.2 M CH_3_COONa, 0.2 M CH_3_COOH, 0.15 mM NaCl, pH 6.0) were added into a 96-well plate. The 0.85% saline solution was used as a negative control. Incubation occurred at 37 °C for 30 min, and after this period, 200 μL of 2.5% CTAB and 2% NaOH solution (CTAB: Cetyltrimethylammonium bromide) were added and incubated for 10 min at ambient temperature. The measurement was carried out on an Epoch plate reader at 405 nm. The HYA activity was expressed as a percentage of turbidity lost, where higher hyaluronidase activity consumes more hyaluronic acid, reducing the turbidity of the medium.

Phospholipase A_2_ (PLA_2_) activity was measured as previously described by Holzer and Mackessy [38]. In a 96-well plate, were added 20 μL of venom diluted in 0.85% saline (concentration 1 mg/mL), 20 μL of deionized water, 20 μL of NOBA substrate (4-nitro-3-(octanoloxy) benzoic acid), 0.32 mM diluted in acetonitrile, and 200 μL of Tris buffer (10 mM, 10 mM CaCl_2_, 100 mM NaCl). The 0.85% saline solution was used as a negative control. Incubation occurred at 37 °C for 60 min with activity measurements every 15 min. The reading was made on an Epoch plate reader at 425 nm. The PLA_2_ activity was expressed in U/min/mg, meaning the quantity of chromophore released per incubation time per mg of protein. An increase of 0.1 unit (U) is equivalent to the release of 25.8 nmol of chromophore.

The proteolytic activity of *Lachesis muta* venom was determined as described by Antunes [39], with modifications. Ten μL of venom diluted in 0.85% saline (concentration 1 mg/mL), 10 μL of 0.85% saline, and 85 μL of azocasein substrate diluted in 50 mM Tris-HCl buffer, 5 mM CaCl_2_, 150 mM NaCl, pH 8.0 were pipetted into microtubes. The 0.85% saline solution was used as a negative control. This mixture was incubated at 37 °C for 30 min. After incubation, 200 μL of 5% TCA (trichloroacetic acid) was added to stop the reaction, and the samples were centrifuged at 1700× *g* for 5 min at 4 °C. Then, 100 μL of the supernatant was applied to a 96-well plate with 100 μL of 0.5 M NaOH. The reading was taken on an Epoch plate reader at 450 nm. Proteolytic activity was expressed in U/min/mg, which is units per incubation time per mg of protein, where an increase in one unit (U) is equivalent to an increase of 0.005 absorbance units.

### 2.5. Biological Activities

The minimum coagulant dose (MCD) is defined as the quantity of protein necessary to coagulate a standard volume of plasma in 60 s at 37 °C, expressed in µg venom/mL plasma. The methodology was based on Theakston and Reid [40]. Incubation of 100 μL human plasma (CAAE process no.: 77262423.3.0000.9487) with 50 μL CaCl_2_ was conducted for 2 min before adding 50 μL of *L. muta* venom diluted in 0.85% saline at different concentrations. The coagulation time is recorded immediately after the addition of the venom on the MaxCoag coagulometer (MEDMAX, São Paulo, Brazil).

Indirect hemolysis activity was performed according to Sæbø [41], with modifications. To perform the test, a 1% human red blood cell solution was prepared with a 0.25% phospholipid solution, 10 mM CaCl_2_ in PBS (phosphate-buffered saline). The venoms were diluted in 0.85% saline solution at different concentrations. In a microtube were added 250 μL of diluted venom and 250 μL of the 1% red blood cell solution. This mixture was incubated at 37 °C for 60 min and then centrifuged at 2000 rpm for 5 min. The supernatant (100 μL) was transferred to a microplate and read on the Epoch plate reader at 405 nm. Hemolytic activity was expressed as effective concentration (EC_50_), which is the required quantity, in µg, of venom to promote the lysis in 50% of erythrocytes.

### 2.6. Statistical Analyses

Statistical analyses were performed using GraphPad Prism 9 software, applying One-Way ANOVA for enzymatic and biological activity results, Two-Way ANOVA for indirect ELISA, and EC_50_ for indirect hemolysis. Statistical differences were considered significant when *p* ≤ 0.05.

## 3. Results

**Figure 1:**
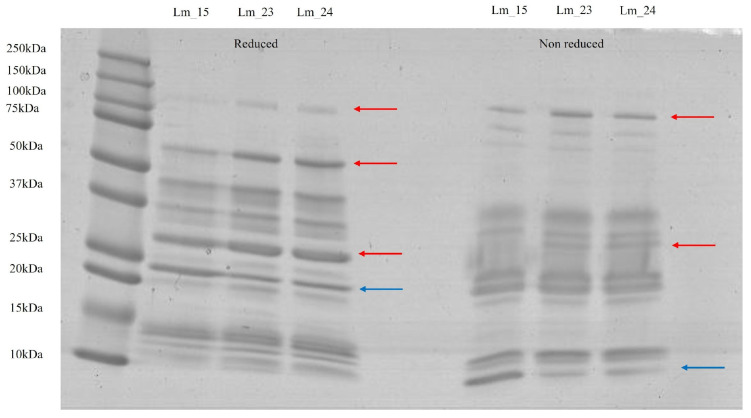
Protein profile by SDS-PAGE in polyacrylamide gel in gradient (10 to 20%) of *Lachesis muta* samples with β-mercaptoethanol (reduced) and without β-mercaptoethanol (non-reduced). The arrows indicate variation in band intensity from (**left**) (Lm_15) to (**right**) (Lm_24), where red arrows represent an increase in intensity and blue arrows represent a decrease in intensity.

Since the samples belonged to the same individual, the protein profile observed in the SDS-PAGE exhibited high homogeneity (Figure 1). The bands were visible between approximately 10 kDa and 130 kDa. Under reduced conditions, a band of 23 kDa was more intense for Lm_15 compared to the other samples. However, in three regions (30 kDa, 60 kDa, and 130 kDa), the bands intensified in Lm_23 and Lm_24. In the samples without β-mercaptoethanol, more pronounced bands could be observed in Lm_15 in the low-molecular-mass region, around 10 kDa. Although less intense bands for this sample occurred at 30 kDa and 130 kDa in comparison to the Lm_23 and Lm_24 samples.

**Figure 2:**
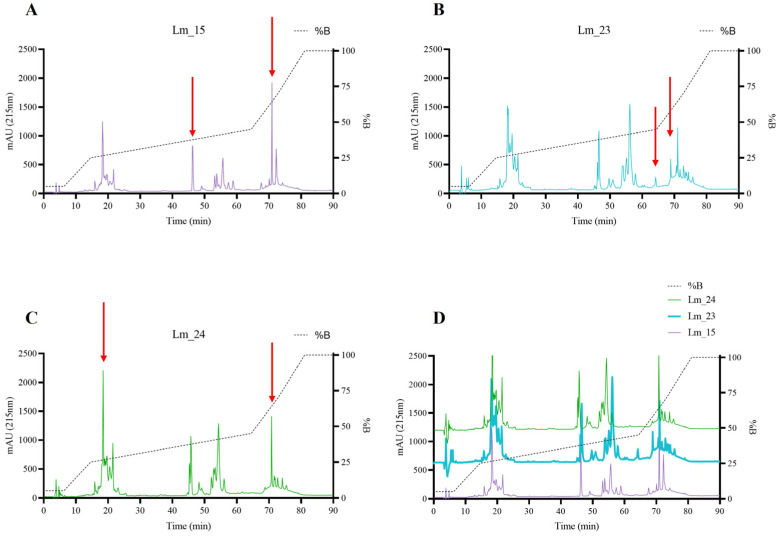
Chromatographic profile of samples Lm_15 (**A**; purple), Lm_23 (**B**; blue), Lm_24 (**C**; green), and overlapped samples (**D**). The samples were subjected to reversed-phase high-performance liquid chromatography (RP-HPLC) on a C18 25 × 0.04 m column with a gradient elution of 95% acetonitrile and 0.1% trifluoroacetic acid. The red arrows highlight the differential peaks between the samples. The absorbance of the peak intensity was measured at 215 nm.

The chromatographic profiles of the three samples were also very similar to one another, with subtle differences; however, in general, Lm_15 showed lower peak intensities (Figure 2). In the initial region (between 10 and 30 min), an increase in peak intensity could be observed throughout the samples, with Lm_15 exhibiting lower peaks, followed by Lm_23 and, finally, Lm_24 with a very intense peak at minute 18. In addition, Lm_23 and Lm_24 showed overlapping peaks between minutes 18 and 22. Another difference occurred from minute 45 onwards, where Lm_15 had a single peak while Lm_23 and Lm_24 had more intense and overlapping peaks followed by double peaks. Lm_23 had an exclusive peak at 64 min of elution. In the final region of the chromatogram, between 68 and 78 min, the samples could also be differentiated. Lm_15 had two main peaks, with the eluate at 72 min being the most intense in all chromatograms, while Lm_23 and Lm_24 had smaller, well-resolved peaks. Lm_23 had a peak at 69 min, not observed in Lm_24, while the peak at 71 min was significantly more intense in the sample from the dead animal.

**Figure 3:**
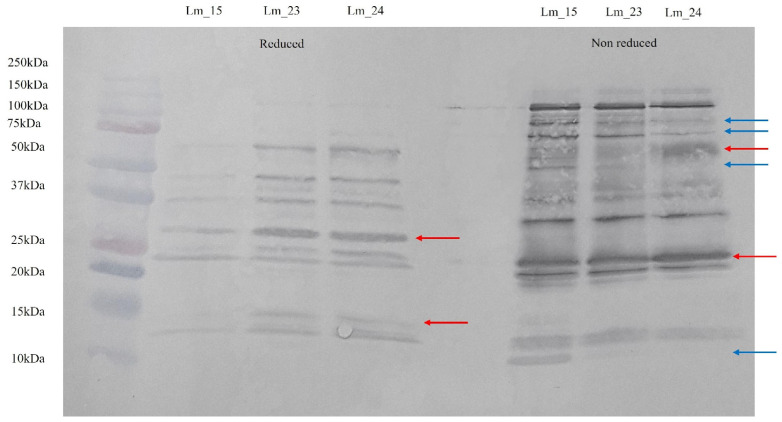
Western blotting performed with anti-*Bothrops*/*Lachesis* serum (ABLS) on a PDVF membrane based on polyacrylamide gel in gradient (10 to 20%). The immunoassay was carried out with β-mercaptoethanol (reduced) and without β-mercaptoethanol (non-reduced). The arrows indicate variation in band intensity from (**left**) (Lm_15) to (**right**) (Lm_24), where red arrows represent an increase in intensity and blue arrows represent a decrease in intensity.

The immunoassays were performed with the anti-*Bothrops*/*Lachesis* serum (ABLS), and all samples were immunorecognized. Western blotting, as well as protein electrophoresis, showed a well-conserved profile among the samples (Figure 3). The reduced samples were less immunologically recognized than the ones without β-mercaptoethanol. More intense bands were visible in Lm_23 and Lm_24 in comparison to the first venom extraction of the animal; examples of this observation were the bands with molecular masses of 15 kDa and 30 kDa. In the non-reduced condition, it was possible to observe the loss of intensity of the bands over the lifetime, where Lm_15 had intense bands, followed by Lm_23 and, lastly, Lm_24. This pattern was seen for the bands in 10 kDa, 55 kDa, 75 kDa, and 100 kDa. However, Lm_24 had more intense bands in two molecular masses: 23 kDa and 60 kDa.

**Figure 4:**
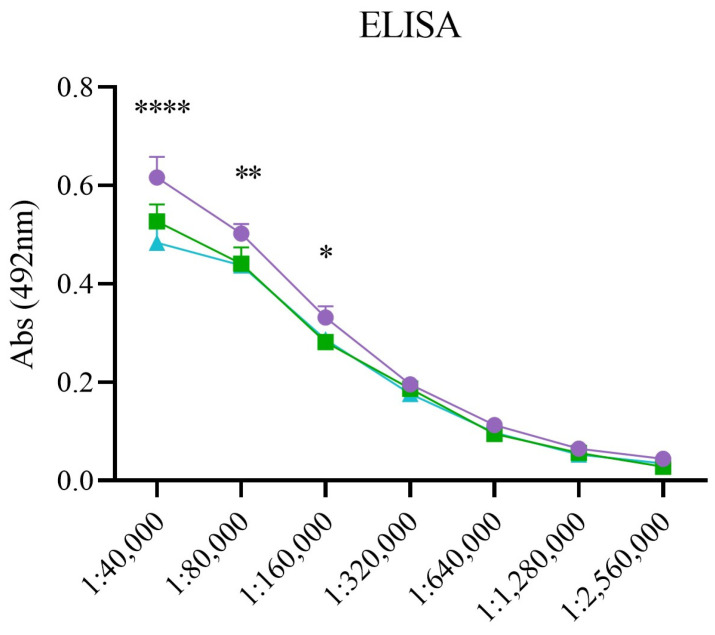
Indirect ELISA immunoassay performed with anti-*Bothrops*/*Lachesis* serum (ABLS). The purple circles represent Lm_15, the blue squares represent Lm_23, and the green triangles represent Lm_24. There is a statistically significant difference (marked with an asterisk) when *p* ≤ 0.05 (*), *p* ≤ 0.01 (**), or *p* ≤ 0.0001 (****).

In the ELISA immunoassay, all the samples were immunorecognized, as well as in the Western blotting assay, despite the statistical difference between them. In the first dilutions of the antivenom serum (1:40,000 until 1:160,000), Lm_15 was the most immunorecognized by ABLS, followed by Lm_23 and, lastly, Lm_24 (Figure 4).

**Figure 5:**
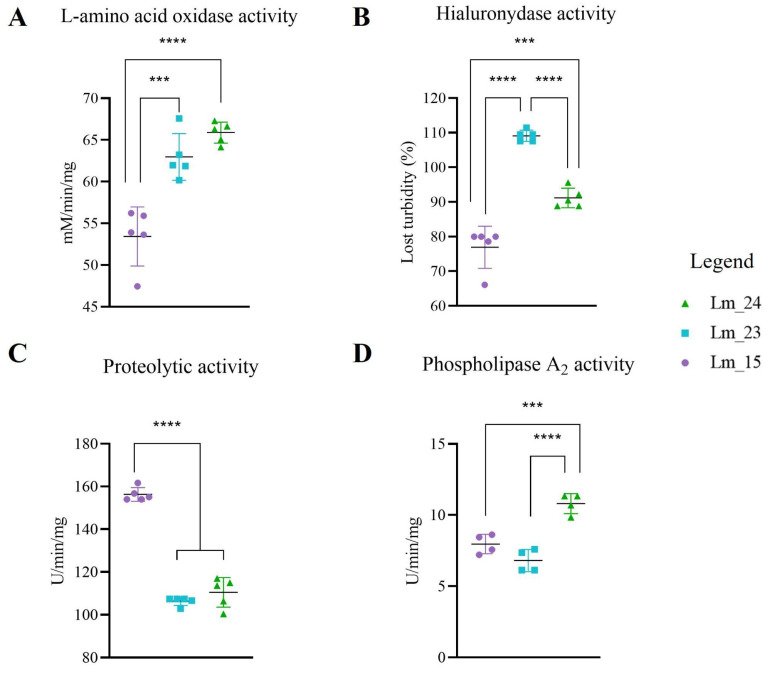
Enzymatic activities of *Lachesis muta* samples, where purple circles represent Lm_15, blue squares represent Lm_23, and green triangles represent Lm_24. The activities were performed with the following substrates: L-amino acid oxidase over L-methionine (LAAO; **A**), hyaluronidase over hyaluronic acid (HYA; **B**), proteolytic activity over azocasein (**C**), and phospholipase A_2_ over NOBA (4-nitro-3-(octanoloxy) benzoic acid) (PLA_2_; **D**). The tests were performed in quintuplicate, and there is a statistically significant difference (marked with an asterisk) when *p* ≤ 0.001 (***), or *p* ≤ 0.0001 (****).

All the enzymatic activities presented statistical differences among the samples (Figure 5; Table 1). The L-amino acid oxidase cleaves L-amino acids, releasing ammonia and hydrogen peroxide; its activity was measured based on the release of H_2_O_2_. The sample Lm_24 exhibited the highest LAAO activity (65.88 mM/min/mg), followed by Lm_15 with 53.43 mM/min/mg and, lastly, Lm_23 with 52.96 mM/min/mg. The same pattern was observed for phospholipase A_2_ (PLA_2_), where Lm_24 had more enzymatic activity (10.8 U/min/mg), as it was the sample that cleaved the most of the synthetic chromogenic substrate NOBA, followed by Lm_15 (7.96 U/min/mg) and Lm_23 (6.8 U/min/mg). In the proteolytic assay, the ability of proteases, serine proteases, and metalloproteinases, to cleave azocasein was measured. Regarding the proteolytic assay, Lm_23 displayed the lowest activity (106.34 U/min/mg), Lm_24 was able to cleave slightly more substrate (110.45 U/min/mg), and Lm_15 was the most proteolytic among the samples (156.26 U/min/mg). Hyaluronidase (HYA) activity also exhibited variations. This assay measures the venom’s ability to degrade hyaluronic acid, reducing the turbidity of the medium. A decrease in turbidity was observed in 76.9% for Lm_15, 91.17% for Lm_24, and 109.1% for Lm_23.

**Figure 6:**
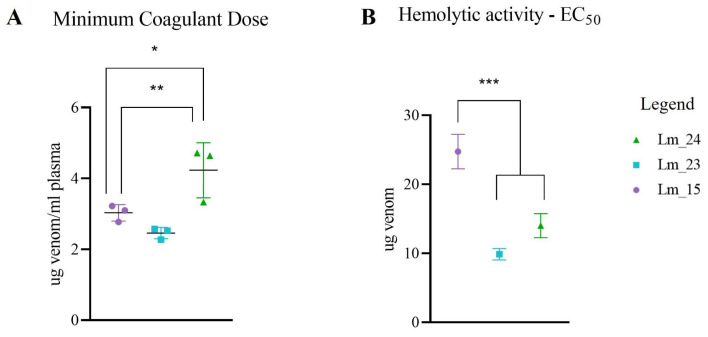
Biological activities of *Lachesis muta* samples, where purple circles represent Lm_15, blue squares represent Lm_23, and green triangles represent Lm_24. The Minimum Coagulant Dose (MCD; **A**) is defined as the quantity of protein necessary to coagulate human plasma in 60 s. Indirect hemolysis (**B**) is expressed in EC_50_, which determines the quantity of venom capable of lysing 50% of red blood cells in solution. The tests were performed in triplicate, and there is a statistically significant difference (marked with an asterisk) when *p* ≤ 0.05 (*), *p* ≤ 0.01 (**), or *p* ≤ 0.001 (***).

As for biological activities, there was also a statistical difference in the assays (Figure 6; Table 2). The minimum coagulant dose establishes the quantity of venom capable of coagulating plasma in 60 s; therefore, lower values indicate greater coagulant capacity of the venom. For the samples evaluated, 3.03 μg venom/mL plasma, 2.457 μg venom/mL plasma, and 4.23 μg venom/mL plasma were required to coagulate the plasma in 60 s for Lm_15, Lm_23, and Lm_24, respectively. Indirect hemolysis was expressed in EC_50_, meaning the amount of venom required to cause 50% lysis of erythrocytes. The values obtained for hemolysis were 24.74 μg venom (Lm_15), 9.864 μg venom (Lm_23), and 14 μg venom (Lm_24). Lm_23 was the most coagulant and hemolytic sample compared to the other venoms.

## 4. Discussion

The current understanding of proteins and activities associated with post-mortem snake venom remains scarce in scientific literature. While incidents involving post-mortem animals are known to occur, there is a notable paucity of publications addressing this specific topic [42]. In light of this significant gap in knowledge, the present study was designed to perform a comprehensive comparison of the protein composition, immunorecognition patterns, and the enzymatic and biological activities of *Lachesis muta* venom, both prior to and following the animal’s death.

The post-mortem venom of *Lachesis muta* exhibited a remarkable similarity to the composition of venom samples collected during the organism’s lifespan. It was also observed that the toxins remained biologically and enzymatically active, indicating that this venom possesses the potential to induce envenomation. The conservation of proteins may occur within the venom gland itself, once there is no contact with light or air, or when stored at low temperatures [42,43]. Envenomation through the manipulation of deceased snakes has been reported for the genera *Crotalus*, *Naja*, *Agkistrodon*, *Bungarus*, and *Pseudechis*, including animals preserved in freezers [42,44,45]. These accidents resulted in symptoms similar to those caused by live animals, with some cases leading to death, and others treated successfully with antivenom serum [42,44,46,47,48,49].

Some studies confirmed that the activities of the venom could also be effectively preserved through appropriate storage conditions. Research on the long-term viability of snake venom was conducted for the genera *Crotalus* and *Bothrops*. Russell [50] demonstrated the persistence of lethality and cardiovascular, neurotoxic, and respiratory effects in the venoms of five *Crotalus* species, even after 26 years of storage in a dark and temperature-controlled facility. Hatakeyama [51] evaluated venoms from *Bothrops jararaca* stored for up to 54 years, either freeze-dried or desiccated at −20 °C. The protein profile and biochemical activities were considered well preserved, showing lethal activity in all periods evaluated. This kind of toxin preservation is mainly due to the absence of water (freeze-drying and desiccation) and low temperatures, which create an unsuitable environment for the action of proteases in the venom, which would otherwise have the potential to degrade the toxins they produce. In addition, refrigerated places also prevent the denaturation of protein structures [52]. Therefore, these studies provide further evidence of the possibility of preserving the biochemical and biological activities of a post-mortem snake venom sample such as Lm_24.

Some subtle differences found in the protein profile might result from the death of the animal. In the Lm_24 chromatogram, the peptide elution region had very intense peaks, which might be due to protein fragmentation, as there is also a decrease in peak intensity toward the end of the chromatogram. This fluctuation in peak intensity might be the result of autolytic cleavage by PII-type and PIII-type metalloproteases (reducing the peaks between 70 and 80 min of elution) with the release of the disintegrin and disintegrin-like domain, respectively (increasing the initial peaks in the chromatogram) [53,54]. In addition, the venom still exhibited proteolytic activity and possibly could have affected the toxins themselves. Khalil [55,56] observed an acceleration of the decomposition process in rabbits with the administration of *Naja haje* and *Cerastes cerastes* venom, highlighting the proteolytic role these toxins play in organic matter [57]. However, if proteolysis had occurred with Lm_24, this would not have been limited to only certain proteins, but instead would have affected all proteins, resulting in significantly less intense peaks in the mid (PLA_2_ and SVSP) and final (SVMP and LAAO) regions of the chromatogram and a higher concentration of bands in the SDS-PAGE in the low molecular mass region. Although the Lm_24 sample was liquid and at room temperature inside the venom gland, increasing the susceptibility of protein degradation by environmental factors such as temperature, proteolysis could not be confirmed. Despite the decrease in proteolytic and hyaluronidase activity, an increase in PLA_2_ and LAAO activity was also observed (Figure 5), thereby invalidating this hypothesis.

Another factor that likely contributes to subtle differences in protein profile is age. Variations between young and adult *Lachesis* have already been reported in the literature, with adults being more proteolytic than juveniles [1,11,28]. In a study with *B. moojeni*, proteolytic activity decreases in senile individuals compared to adults [30]. Therefore, age might have influenced proteases, since Lm_23 and Lm_24 (when the snake was older) showed a similar pattern in the chromatogram for SVSP and SVMP-PI (between 50 and 60 min of elution) with more intense peaks than Lm_15 (Figure 2), except in the SVMP-PIII peak (approximately at 72 min), more intense in the adult animal sample (Lm_15) [13,14], corroborating the azocasein assay (Figure 5) in which this individual presented at least 29% more proteolytic activity than the other two samples. The protein profile in SDS-PAGE was also very similar between Lm_23 and Lm_24. In fact, both samples appeared to undergo the same increases and decreases in band intensity (as indicated by the red and blue arrows, respectively, Figure 1). Therefore, age seems to have a greater impact than death on these samples, since the difference between Lm_15 and the other samples was at least eight years, while between Lm_23 and Lm_24 there was only a ten-month difference, suggesting that the characteristics exhibited by these samples were more closely linked to age.

L-amino acid oxidase was likely affected by the animal’s death. As a thermolabile enzyme, LAAO is inactivated at extreme temperatures, both high and low, with optimal activity between 0° and 50 °C. Ideally, samples with LAAO should be stored at 4 °C at neutral pH, avoiding freezing and thawing cycles [58,59,60]. The samples employed in this study were stored for different storage periods, which might have contributed to the differences in activity observed in this assay, since for the species *L. muta*, the enzyme loses activity when subjected to temperatures of −70 °C, −20 °C, and 100 °C [61]. Lm_24 was frozen for only a few weeks and was the sample with the highest LAAO activity (Figure 5). Lm_24 had approximately 19% more LAAO activity in comparison to the other samples. Associated with the enzymatic assay, the post-mortem sample showed intense bands in SDS-PAGE with an approximate molecular mass of 65 kDa (with β-mercaptoethanol) and 130 kDa (without β-mercaptoethanol) (Figure 1), consistent with LAAO [59,60]. Therefore, post-mortem analysis of the venom allowed for higher detection of LAAO activity in the venom of *Lachesis muta*. In addition, age may also have influenced the variation in enzyme activity. In a study with *B. mattogrossensis*, LAAO activity increased along with the aging of the individuals analyzed, being highest in senile snakes [31], which corroborates our results.

Phospholipase A_2_ activity also appears to be influenced by post-mortem status, considering the statistical difference between samples from the live snake (Lm_15: 7.96 U/mg/min and Lm_23: 6.8 U/mg/min) compared to the post-mortem sample (Lm_24: 10.8 U/mg/min). Although no definitive hypothesis can be proposed for the increase in PLA_2_ activity in the Lm_24 sample, the characteristics of this protein—which is highly stable due to its 6 to 8 disulfide bridges—suggest that phospholipase A_2_ was able to maintain structure and activity even after the surucucu’s death [62]. Literature indicates other factors that can cause variation in PLA_2_ activity. Studies on ontogeny reveal that phospholipase A_2_ shows greater activity in younger individuals, as seen for *B. jararaca*, *B. erythromelas*, *B. jararacussu*, and *B. moojeni* [18,26,30,39], whereas older individuals exhibit higher phospholipase A_2_ activity for *B. mattogrossensis* [31]. In the *Bothrops* genus, males also tend to exhibit higher PLA_2_ activity than females among the species *B. mattogrossensis*, *B. jararacussu*, *B. pauloensis*, and *B. erythromelas* [18,26,27,31]. Such studies demonstrate an increased demand for further research on factors that may influence the variation in phospholipase A_2_ activity within the *Lachesis* genus.

Biological activities usually result from the synergistic action of venom toxins [63,64], a property that persists even after the animal’s death. The MCD is highly influenced by proteases (SVSP and SVMP), with procoagulant potential [9,57,65,66], whilst PLA_2_ promotes an anticoagulant profile of the venom [67,68]. Therefore, the combination of these toxins results in the group of symptoms observed in *Lachesis* accident, where initially there is a delayed coagulation followed by hemorrhage [3,6,7,8]. Despite being less coagulant than the other samples, Lm_24 still exhibited coagulant properties within the expected range for the genus, since a study with several samples of *L. muta* and *L. rhombeata*, the MDC was determined between 8.7 and 2.5 μg venom/mL plasma [12]. Similarly, hemolysis is also due to the action of more than one toxin; however, PLA_2_ is primarily responsible [69,70,71,72]. Studies indicate that LAAO, C-type lectins, and SVSP also participate in the process of erythrocyte hemolysis [56,73,74,75,76]. Considering that Lm_24 displayed higher PLA_2_ and LAAO enzymatic activities than the other samples, it was expected to exhibit an increased hemolytic activity, even though from a deceased animal.

Despite variations in protein profile and toxin activity, all samples were immuno-recognized in indirect ELISA and Western blotting by ABLS, including Lm_24, derived from a deceased animal (Figure 3 and Figure 4). In Brazil, antivenom serum is obtained by administering a sublethal dose of adult snake venom to horses so their immune systems produce antibodies. The F(ab′)2 fractions of heterologous immunoglobulins specific against venom toxins are then purified to constitute the antivenom serum [32]. Considering the context of envenomation, identifying the immune recognition of venoms from senile and dead snakes by antivenom is very relevant because this indicates whether this medicine can be used to treat accidents involving these snakes. The venoms from senescent snakes of the species *B. leucurus*, *B. moojeni*, and *B. mattogrossensis* were also immunologically recognized by antivenom serum [29,30,31]. Additionally, as previously mentioned, patients bitten by dead snakes were successfully treated with serotherapy [44,46,48,49]. Considering the persistence of biochemical activities and, mostly, the immune recognition of Lm_24, the venom of dead snakes could be an option to produce antivenom serum, in extreme cases. Although it is not the ideal situation, as the recommended practice would be using the venom of healthy adult animals [30,39], it is nevertheless necessary to consider alternatives in case of a shortage of venom supplies, especially considering the difficulty of maintaining *Lachesis* ex situ [3,10]. Also, Butantan Institute, the main producer of antivenom in Brazil, reported a decrease in donations and snake captures in the last few decades [15]. The proposal to use venom from senile animals for serum production has already been raised in a study with *B. moojeni* [30], as well as venom from *Bothrops spp.* with liver tumors [77].

The subtle variations found in the venom of *Lachesis muta* appear to be more related to the senility of the animal than to death. However, due to the small sample size (three samples from a single individual), we cannot conclusively determine the origin of these variations. Our study group has been evaluating the impact of ontogeny and senility in several Brazilian venomous snake species of the genera *Bothrops* and *Crotalus*, such *as B. leucurus*, *B. jararacussu*, *B. erythromelas*, *B. moojeni*, *B. pauloensis*, and *C. durissus* [18,24,25,26,27,29,30,31], confirming that the venom of the Viperidae family can undergo variations according to age.

## 5. Conclusions

The venom of *Lachesis muta* presented increased enzymatic activities of L-amino acid oxidase and phospholipase A_2_ and decreased coagulation capacity post-mortem. Despite the variations found, the biochemical activities were preserved, along with the protein composition of this sample. It is believed that these variations are more likely due to the difference in age between the venoms analyzed. More importantly, the venom of a deceased animal was immunologically recognized by ABLS. Therefore, based on the data presented in this preliminary study, in emergency cases, the venom of a dead snake could be included in the venom pool designated to produce antivenom serum.

## Figures and Tables

**Table 1:** Enzymatic activities from the *Lachesis muta* samples.

Enzymatic Activity	Lm_15	Lm_23	Lm_24
L-aminoacid oxidase (mM/min/mg) ^a,c^	53.43 ± 3.54	62.96 ± 2.8	65.88 ± 1.27
Hyaluronidase (% Lost turbidity) ^a,b,c^	76.90 ± 6.07	109.1± 1.62	91.17 ± 2.82
Proteolytic (azocasein) (U/min/mg) ^a,c^	156.26 ± 3.19	106.34 ± 1.98	110.45 ± 6.91
Phospholipase A_2_ (U/min/mg) ^b,c^	7.96± 0.68	6.8 ± 0.78	10.8 ± 0.71

Statistical differences were considered significant when *p* < 0.05. The letters indicate this variation between groups, where ‘^a^’ represents differences between Lm_15 and Lm_23; ‘^b^’ indicates differences between Lm_23 and Lm_24; ‘^c^’ stands for differences between Lm_15 and Lm_24.

**Table 2:** Biological activities from the *Lachesis muta* samples.

Biological Activity	Lm_15	Lm_23	Lm_24
Minimum coagulant dose (ug venom/mL plasma) ^b,c^	3.03 ± 0.23	2.457 ± 0.161	4.23 ± 0.778
Hemolysis (ug venom) ^a,c^	24.74 ± 1.45	9.864 ± 0.473	14 ± 1

Statistical differences were considered significant when *p* < 0.05. The letters indicate this variation between groups, where ‘^a^’ represents differences between Lm_15 and Lm_23; ‘^b^’ indicates differences between Lm_23 and Lm_24; ‘^c^’ stands for differences between Lm_15 and Lm_24.2.3.

## Data Availability

Data is contained within the article.
